# Sevoflurane exposure induces neurotoxicity by regulating mitochondrial function of microglia due to NAD insufficiency

**DOI:** 10.3389/fncel.2022.914957

**Published:** 2022-09-21

**Authors:** Ruilou Zhu, Shuang Zeng, Ningning Li, Ningning Fu, Yangyang Wang, Mengrong Miao, Yitian Yang, Mingyang Sun, Jiaqiang Zhang

**Affiliations:** Department of Anesthesiology and Perioperative Medicine, Center for Clinical Single Cell Biomedicine, Henan Provincial People’s Hospital, People’s Hospital of Zhengzhou University, Zhengzhou, China

**Keywords:** sevoflurane, neurotoxicity, microglia, mitochondria, NAD

## Abstract

Developmental neurons received with sevoflurane, the commonly used inhalational anesthetic agent in clinical surgery, several times tend to be destroyed. Microglia, the resident immune cells of the central nervous system (CNS), are activated after sevoflurane exposure, accompanied by releasing proinflammatory cytokines that damage developing neurons. The sevoflurane-induced neurotoxicity could be attributed to activated microglia presenting proinflammatory and anti-inflammatory functions. Proinflammatory microglia release cytokines to impair the CNS, while anti-inflammatory microglia engulf damaged neurons to maintain CNS homeostasis. Sevoflurane exposure promotes the secretion of proinflammatory cytokines by microglia, inhibiting the microglial phagocytic function. Microglia with poor phagocytic function cannot engulf damaged neurons, leading to the accumulation of damaged neurons. The mechanism underlying poor phagocytic function may be attributed to mitochondrial dysfunction of microglia induced by sevoflurane exposure, in which affected mitochondria cannot generate adequate ATP and NAD to satisfy the energy demand. We discovered that sevoflurane treatment impaired the mitochondrial metabolism of microglia, which resulted in NAD deficiency and couldn’t produce sufficient energy to clear damaged neurons to maintain CNS development. Our findings provide an explanation of a new mechanism underlying sevoflurane-induced neurotoxicity.

## Introduction

Sevoflurane, the commonly used inhalational anesthetic agent, is widely used in neonatal and pediatric surgery due to its rapid induction and recovery profile (Eger, 1994). However, continual exposure to sevoflurane leads to neuronal damage in neonatal rodents (Jevtovic-Todorovic et al., 2003). The underlying mechanisms of sevoflurane-induced neuronal apoptosis are still unknown. Neuro-inflammation, including proinflammatory cytokine accumulation and microglia activation, which has been known to be involved in neuron degeneration, may contribute to neuro-apoptosis after sevoflurane exposure (van Gool et al., 2010; Shen et al., 2013).

Microglia, the macrophages in the central nervous system (CNS) accounting for 10% of the cells within the CNS, can alter their morphology to sustain brain homeostasis (Aguzzi et al., 2013). In the healthy CNS, ramified microglia characterized by delicate branching processes perform immune surveillance. Microglia are activated after being attacked. Then, the cell body extends in size, the proximal processes become thicker, and the branches are less ramified. However, activated microglia are double-edged swords because they not only release proinflammatory mediators as classically activated microglia but also release numerous protective and trophic factors as alternatively activated microglia (Mosser and Edwards, 2008; Pozzo et al., 2019). A previous study reported that sevoflurane exposure causes the activation of microglia and releases proinflammatory factors, such as TNFα, IL-6, and IL-1α, which may dampen early brain development (Shen et al., 2013). We, therefore, hypothesized that sevoflurane exposure induces microglia to perform proinflammatory function, which is detrimental to developing neurons, and that sevoflurane exposure also inhibits the anti-inflammatory function of microglia.

Microglia are identical to other immune cells in that the polarization to the classically activated phenotype is often accompanied by declined oxidative phosphorylation (Orihuela et al., 2016). However, proinflammatory microglia impair mitochondrial function, leading to insufficient energy (Orihuela et al., 2016). Activated microglia of the alternatively activated phenotype can engulf damaged neurons to protect the CNS from attack (Brown and Neher, 2014). Furthermore, reports have indicated that increased oxidative phosphorylation is a hallmark of the alternatively activated phenotype (Vats et al., 2006; Rodriguez-Prados et al., 2010). The initiation and maintenance of phagocytosis are dependent on accelerated glycolytic and mitochondrial metabolism to satisfy energy demand (Pearce and Pearce, 2013; Mills et al., 2017). Cognitive dysfunction induced by sevoflurane exposure in young mice was alleviated when treated with CoQ10, a mitochondrial function enhancer (Xu et al., 2017). This evidence suggests that the mitochondrial function is critical for regulating the microglia phenotype.

Previous studies show that NAD^+^ is critical for mitochondrial metabolism (Katsyuba and Auwerx, 2017). The cofactor NAD^+^ is essential for both glycolysis and oxidative phosphorylation, feeding electrons into complex I of the electron transport chain to drive oxidative phosphorylation. NAD^+^ boosting helps to improve mitochondrial dysfunction (Romani et al., 2021). A study demonstrated that restoration of NAD^+^ levels could alleviate neuronal damage (Mukherjee et al., 1997). P7C3, the NAD^+^ agonist, can enhance neurogenesis (Alano et al., 2004) and can be neuroprotective against various neurodegenerative diseases (Pieper et al., 2010; De Jesus-Cortes et al., 2012). These proofs suggest that NAD^+^ is essential for mitochondrial function and neuronal degeneration protection.

In this article, we discovered that microglia activation was involved in sevoflurane-induced neurotoxicity by impairing mitochondrial function through affecting NAD^+^ content. Our study demonstrated that microglia were activated after sevoflurane exposure, releasing proinflammatory factors while inhibiting phagocytic functions. The upregulated proinflammatory cytokines released by the microglia were detrimental to neurons. Furthermore, sevoflurane exposure restricted the mitochondrial function of microglia, and both ATP production and NAD^+^ generation were declined. Thus, the microglia could not produce adequate ATP to remove damaged neurons, resulting in neuronal apoptosis. Treatment with NAD^+^ mitigated mitochondrial dysfunction and improved phagocytic function of microglia, which demonstrated NAD^+^ insufficiency contributed to neuron degeneration induced by sevoflurane exposure. Our work uncovers a new mechanism underlying sevoflurane-induced neurotoxicity and sheds new light on therapeutic targets for neuronal degeneration treatment.

## Materials and methods

### Mice, anesthesia, and treatment

All of the animal experiments were approved by the Henan Provincial People’s Hospital Institutional Animal Care and Use Committee. All mice were housed in an environmentally controlled, pathogen-free barrier facility on a 12-h light–dark cycle, temperature, and humidity, with food and water available *ad libitum*. The C57BL/6J background mice were purchased from GemPharmatech (Nanjing). The mice of both sexes were treated with 3% sevoflurane plus 30% oxygen for 2 h on postnatal days 6, 7, and 8. We continuously monitored concentrations of sevoflurane and oxygen using a gas analyzer (Drager, Germany) during anesthesia administration. The anesthesia chamber, obtained from STEMCELL Technologies (Vancouver, Canada), was heated *via* a warming pad placed under this chamber to keep the mouse rectal temperature at 37 ± 0.5°C.

### Cell culture and RNA sequencing

BV-2 cell lines were cultured in the DMEM medium containing 10% FBS (Gibco, America) with penicillin (100 U/ml) and streptomycin (100 mg/ml). The cells were maintained in a humidified atmosphere that contained 5% CO_2_ at 37°C. RNA sequencing (RNA-seq) and bioinformatic analysis were performed by Beijing Genomics Institute (BGI, Beijing, China).

### Tissue sample collection and RNA extraction

The cortex was harvested immediately after cervical dislocation. BV-2 cell line was harvested with 0.25% trypsin-EDTA (Solarbio Life Science, Beijing, China). Total RNA was isolated from the cortex and BV-2 cell line using TRIzol reagent (Takara), and the cDNA was synthesized with the PrimeScript™ RT Master Mix (Takara) according to the manufacturer’s protocol. Quantitative PCR was performed with the SYBR™ Select Master Mix (Applied Biosystems, United States) using the Applied Biosystems Real-Time PCR system. The relative mRNA level values were normalized to β-actin to calculate fold changes in expression. The primer sequences used in this study are listed in Table 1.

**TABLE 1:** Primer list.

Gene symbol	Forward primer (5′–3′)	Reverse primer (5′–3′)
β-actin	GGCTGTATTCCCCTCCATCG	CCAGTTGGTAACAATGCCATGT
IL-10	GCTCTTACTGACTGGCATGAG	CGCAGCTCTAGGAGCATGTG
TNFα	CCCTCACACTCAGATCATCTTCT	GCTACGACGTGGGCTACAG
IL-6	TAGTCCTTCCTACCCCAATTTCC	TTGGTCCTTAGCCACTCCTTC
IL-1α	CGAAGACTACAGTTCTGCCATT	GACGTTTCAGAGGTTCTCAGAG
IL-1β	GCAACTGTTCCTGAACTCAACT	ATCTTTTGGGGTCCGTCAACT
Aif1	ATCAACAAGCAATTCCTCGATGA	CAGCATTCGCTTCAAGGACATA

### Enzyme-linked immunosorbent assay, NAD^+^ concentration detection, and ATP concentration detection

We homogenized the harvested cortex and BV-2 cells on ice using RIPA buffer supplemented with protease inhibitors. The lysates were centrifuged at 12,000 rpm for 10 min at 4°C. The supernatant was collected to detect the protein concentration by enzyme-linked immunosorbent assay (ELISA) (Neobioscience, Beijing, China). The experiment was conducted following the operation manual. Briefly, the standard and protein samples were added to the reaction wells of the 96-well plate and were incubated at 37°C for 90 min. Then, each well was aspirated and washed 5 times, and a biotinylated antibody was added and then incubated at 37°C for 60 min. After washing each well 5 times, enzyme conjugate was added to each well and incubated at 37°C for 30 min protecting it from light. Chromogenic substrate was added to each well and incubated at 37°C for 15 min, followed by the addition of a stop solution, and a microplate reader set to 450 nm was used.

We used NAD^+^ production assay (Beyotime Biotechnology, Shanghai, China) to detect NAD^+^ concentration. In addition, the cell lysis was obtained by NAD^+^ extraction, and then, the samples were centrifuged at 12,000 rpm for 10 min at 4°C. The supernatant was collected; alcohol dehydrogenase was added to the supernatant and then incubated for 10 min at 37°C avoiding light. Next, the chromogenic agent was added to each sample and incubated for 30 min at 37°C, followed by using a microplate reader set to 450 nm. We used an ATP production assay (Beyotime Biotechnology, Shanghai, China) to detect ATP production, and the sections were conducted following the manufacturer’s protocol.

### Immunofluorescence, TUNEL assays, and phagocytic assay

Mice were transcardially perfused with saline and 50 ml of 4% paraformaldehyde. For immunofluorescence, the brains were dissected and immersed in the same fixative for 4 h at 4°C. The sections (20 μm) were obtained using cryostats and stored at −80°C. All washes and incubations were done in PBS containing 0.3% Triton X-100 (Solarbio Life Science, Beijing, China). Sections were pre-incubated in 5% normal goat serum for 1 h at room temperature, followed by overnight incubation with primary antibody Iba1 (Abcam, 1:200, England) and CD45 (Invitrogen, 1:200, United States) at 4°C. After plentiful washing, sections were incubated with the appropriate secondary antibody conjugated with Alexa488 (1:200) for 1 h at room temperature. After washing, the sections were analyzed on Olympus microscope.

For TUNEL (TdT-mediated dUTP Nick-End Labeling) assay, we performed the experiment using the TUNEL detection kit (Promega, United States), and the sections were conducted following the manufacturer’s protocol. For the phagocytic assay, we treated the BV-2 cell with pHrodo™ Green BioParticles (Thermo Fisher Scientific, United States) for 2 h in a 37°C cell incubator. After washing the cells using PBS two times, the cells were fixed with 4% paraformaldehyde (Biosharp, China) for 15 min, and the cells were analyzed on Olympus microscope.

### Real-time oxygen consumption rate

Cells were plated at 2 × 10^5^ cells per well in a Seahorse XF24 Cell Culture Microplate (Agilent, United States). Cells were washed two times with Agilent Seahorse XF Media (Agilent, United States) supplemented with 1 mM pyruvate, 2 mM L-glutamine, and 2 mM D-glucose; a final volume of 525 μl was placed in each well. Cells were then incubated in a 0% CO_2_ chamber at 37°C for 1 h before being placed into a Seahorse XFe24 Analyzer (Agilent, United States). For OCR experiments, cells were treated with 1.5 μM oligomycin, 1.5 μM carbonyl cyanide p-trifluoromethoxyphenylhydrazone (FCCP), and 0.5 μM rotenone/antimycin. A total of three OCR and pH measurements were taken after each compound was administered. All Seahorse experiments were repeated at least three times.

### Statistical analysis

All data were presented as the mean ± s.e.m. Statistical comparisons were performed with unpaired two-tailed Student’s *t*-test and one-way ANOVA, which were carried out in GraphPad Prism8. In all cases, statistical significance was indicated as *p < 0.05 or ^**^p < 0.01.

## Results

### Sevoflurane led to developing neuronal apoptosis by increasing the expression of inflammatory factors

**FIGURE 1:**
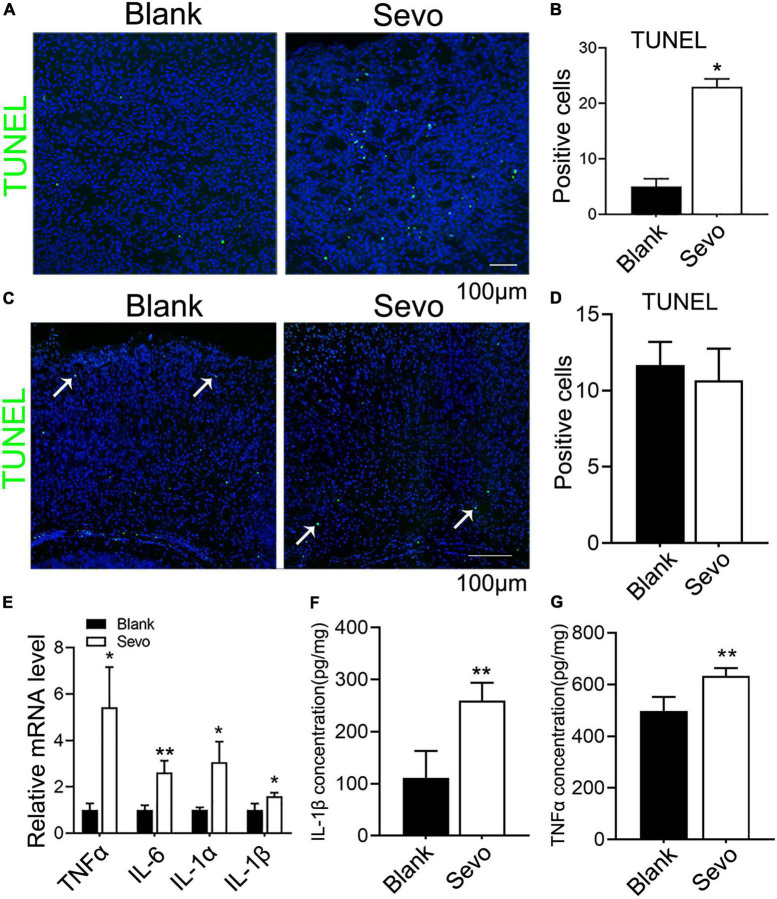
Sevoflurane led to developing neuronal apoptosis *via* aggravating inflammatory factors expression. **(A,B)** TUNEL assay of brain tissue at 6 days mice of sevoflurane exposure for 2 h daily for 3 days and the quantitative analysis of TUNEL-positive cells. *p* = 0.039. *N* = 3. **(C,D)** TUNEL assay of brain tissues at 30 days mice of sevoflurane exposure and the quantitative analysis of TUNEL-positive cells. *p* = 0.54. *N* = 3. **(E)** Quantitative real-time PCR (qRT-PCR) of brain tissue at 6 days mice of control and sevoflurane exposure groups. p(TNFα) = 0.018, p(IL-6) = 0.013, p(IL-1α) = 0.05, p(IL-1β) = 0.043. *N* = 3. **(F,G)** ELISA experiment with IL-1β and TNFα of control and sevoflurane exposure groups. p(IL-1β) = 0.014, p(TNFα) = 0.005. *N* = 4. Scale bar, 100 μm. *P ≤ 0.05, **P ≤ 0.01.

The 6 days postnatal mice were treated with 3% sevoflurane for 2 h daily for a total of 3 days. Then, we obtained brain tissue from the mice to determine whether the neurons were damaged. The TUNEL assay performed on the cortex samples indicated that the number of TUNEL-positive cells significantly increased after sevoflurane treatment (Figures 1A,B). The immunofluorescence staining with neuron marker NeuN, expressed in mice brain cell nuclei of most post-mitotic neurons (Mullen et al., 1992), demonstrated that the number of neurons declined after sevoflurane exposure (Supplementary Figure 1), which indicated neurons underwent apoptosis after sevoflurane exposure. However, the adult mice that received sevoflurane for 30 days did not show an increase in neuronal apoptosis compared to control groups (Figures 1C,D), which was consistent with previous reports (Yu et al., 2020). Substantial proofs indicated that sevoflurane exposure promoted the secretion of proinflammatory factors, and we found that sevoflurane increased the expression of proinflammatory factors, including TNFα, IL-6, IL-1α, and IL-1β (Figure 1E). Subsequently, ELISA demonstrated that IL-1β and TNFα were highly expressed after sevoflurane treatment (Figures 1F,G). Our findings reveal that the brain cells of neonatal mice treated with sevoflurane underwent neuronal apoptosis due to the release of proinflammatory factors.

### Sevoflurane resulted in microglia activation and phagocytic dysfunction

**FIGURE 2:**
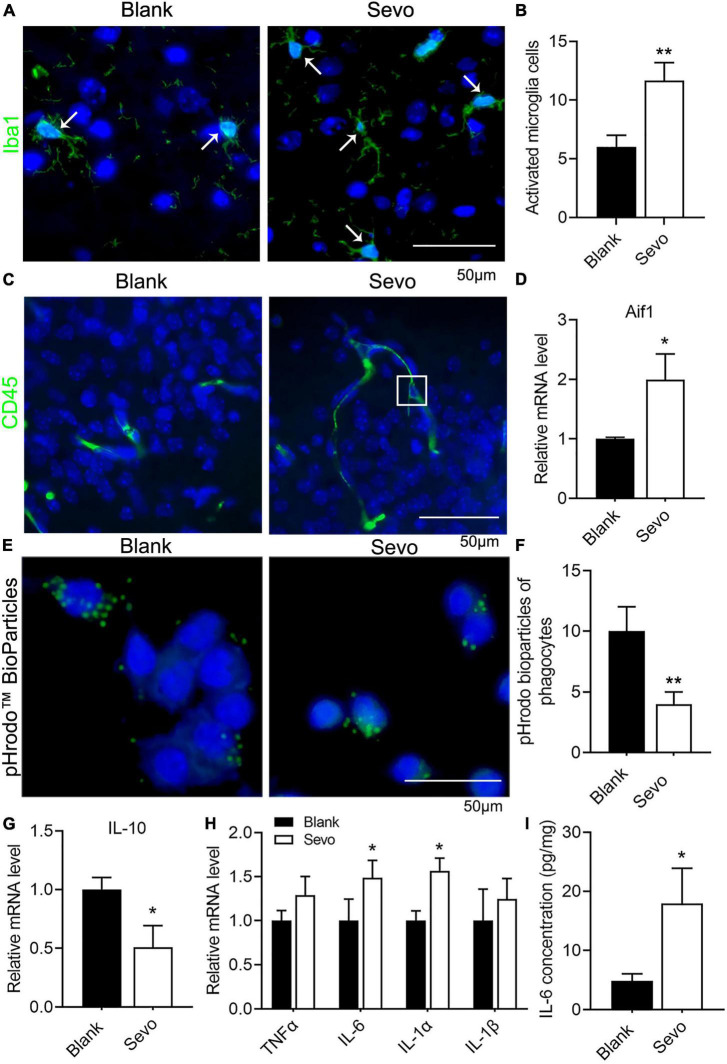
Sevoflurane exposure resulted in microglia activation and phagocytic dysfunction. **(A,B)** Immunofluorescence and quantitative analysis with Iba1 of brain tissue. *p* = 0.013. *N* = 3. **(C)** Immunofluorescence with CD45 of brain tissue. **(D)** qRT-PCR of BV-2 cell line shows the mRNA level of Aif1. *p* = 0.053. *N* = 3. **(E,F)** pHrodo™ Green BioParticles staining and quantification of BV-2 cell line to determine the phagocytic function. *p* = 0.01. *N* = 6. **(G)** qRT-PCR of BV-2 cell line indicates the mRNA level of IL-10. *p* = 0.045. *N* = 3. **(H)** qRT-PCR of BV-2 cell line shows the mRNA level of proinflammatory factors. p(TNFα) = 0.1, p(IL-6) = 0.059, p(IL-1α) = 0.017, p(IL-1β) = 0.39. *N* = 3. **(I)** IL-6 ELISA experiment of BV-2 cell line both in control and sevoflurane group. *p* = 0.036. *N* = 4. Scale bar, 50 μm. *P ≤ 0.05, **P ≤ 0.01.

Microglia are prone to be activated in response to threats. Ionized calcium-binding adaptor molecule 1 (Iba1), expressed in membrane ruffles, is a marker of activated microglia. The immunofluorescence of Iba1 in our samples showed increased expression of Iba1, which suggested that the microglia were activated after sevoflurane treatment for 2 h daily for a total of 3 days (Figures 2A,B). CD45, which outlines the cell body and phagocytic cups of microglia, was also expressed after sevoflurane exposure (Figure 2C). This evidence indicates that the microglia tended to phagocyte apoptotic cells. Next, we treated the microglia cell line BV-2, a well-constructed cell model for microglia cell research (Blasi et al., 1990), with sevoflurane for 24 h and examined the expression of Aif1, also a marker of microglia. The mRNA level of Aif1 was increased after sevoflurane exposure (Figure 2D). To determine whether the phagocytic function of microglia was affected by sevoflurane, we treated the BV-2 cells with pHrodo Green BioParticles and found that the number of BioParticles engulfed by microglia was downregulated after sevoflurane exposure (Figures 2E,F). This test demonstrated that the phagocytic function of microglia was impaired. According to the qRT-PCR results, the expression of the anti-inflammatory factor IL-10 declined (Figure 2G), whereas the expression of proinflammatory factors, including TNFα, IL-6, IL-1α, and IL-1β, significantly increased after sevoflurane exposure (Figure 2H). Additionally, ELISA revealed that the amount of IL-6 secretion by BV-2 cells was upregulated after sevoflurane treatment (Figure 2I). We also checked the TNFα concentration released by microglia using the ELISA experiment and found that TNFα secretion was upregulated after sevoflurane exposure (Supplementary Figure 2). This evidence suggested that sevoflurane exposure led to microglia activation and promotion of inflammatory function while impairing phagocytic function.

### Sevoflurane impaired mitochondrial function and ATP production

**FIGURE 3:**
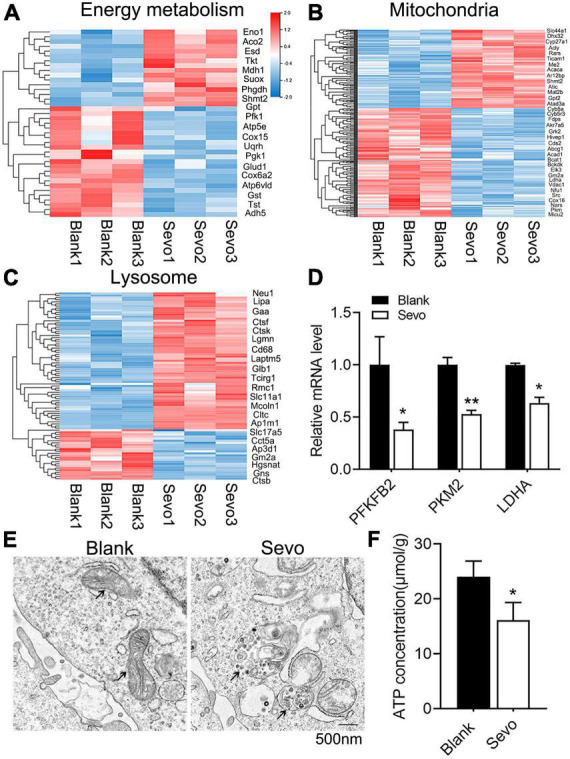
Sevoflurane impaired mitochondrial function and ATP production. **(A–C)** Transcriptome analysis of the RNA-sequence data of BV-2 cell line. The heat map represents the cluster analysis of energy metabolism, mitochondria, and lysosome pathway-associated genes. Expression levels were measured as RPKM-normalized values. Each row represents a differentially expressed gene, while each column represents a sample. The blue and red color gradients indicate decreases and increases in transcript abundances, respectively. **(D)** qRT-PCR of BV-2 cell line shows the mRNA level of metabolism-related genes, including PFKFB2, PKM2, and LDHA. p(PFKFB2) = 0.019, p(PKM2) = 0.01, p(Ldha) = 0.024. *N* = 3. **(E)** TEM of BV-2 cell line, black arrowhead in left image indicates mitochondria, while black arrowhead in the right image indicates the gathered lysosome around the mitochondria. **(F)** ATP production of BV-2 cell line was determined by the ATP production detection kit. *p* = 0.039. *N* = 3. Scale bar, 500 nm. *P ≤ 0.05, **P ≤ 0.01.

To further examine the mechanism involved in the degeneration of microglial phagocytic function, we sequenced the RNA of BV-2 cells to compare the differentially expressed genes of the control and sevoflurane-treated group. The genes involved in energy metabolism, mitochondrial, and lysosomal pathways were altered significantly after sevoflurane exposure (Figures 3A–C). The genes involved in the energy metabolism pathway were downregulated, and genes involved in the mitochondrial pathway were also downregulated, which suggested declined energy production after sevoflurane exposure (Figures 3A,B). Mitochondria–lysosome contacts are involved in regulating mitochondrial function (Wong et al., 2018). Lysosomes, which are filled with digestive enzymes, are recruited to the damaged area of the mitochondria to degrade the dysfunctional organelles. Genes involved in the lysosomal pathway were upregulated after sevoflurane exposure (Figure 3C), indicating lysosomal accumulation. To confirm the RNA-seq results, we checked the expression level of metabolism-associated genes, including PFKFB2, PKM2, and LDHA, and observed decreased mRNA levels of these genes (Figure 3D). The results suggested that the microglial mitochondria may be sensitive to and damaged by sevoflurane exposure.

**FIGURE 4:**
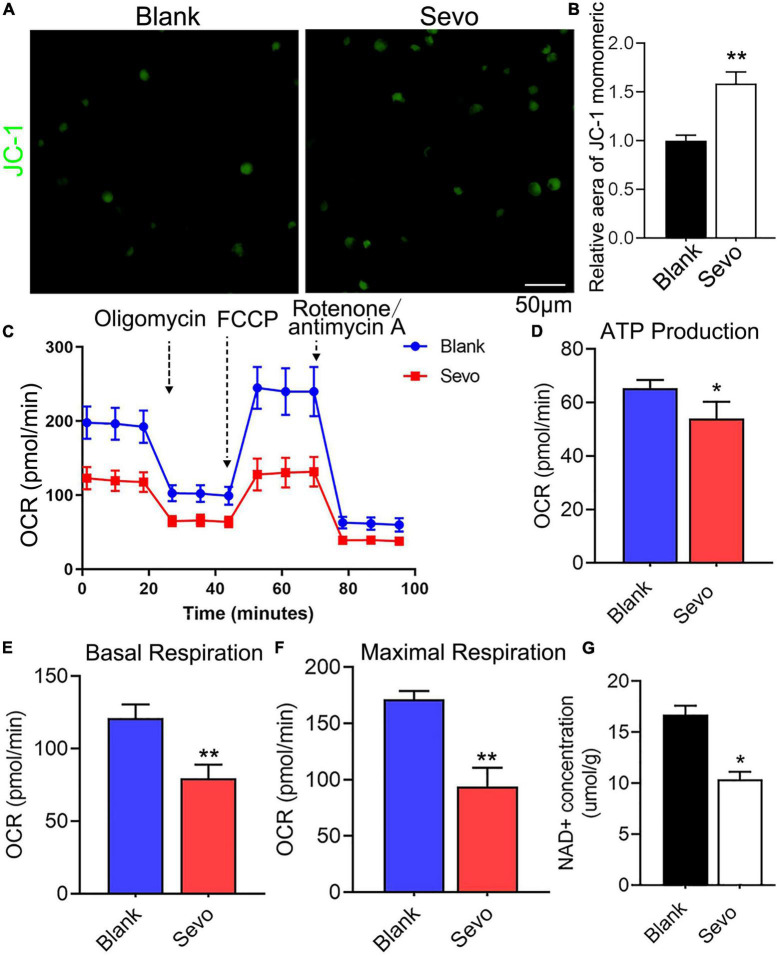
Sevoflurane led to mitochondrial dysfunction. **(A,B)** Staining and quantitative analysis with JC-1 of BV-2 cell line. *p* = 0.0015. *N* = 3. **(C)** The overall mitochondrial metabolism using Seahorse experiment of BV-2 cell line. **(D–F)** The ATP production, basal respiration, and maximal respiration treated with mitochondrial stress kit agent including oligomycin, FCCP, and rotenone/antimycin A. p(ATP production) = 0.022, p(Basal respiration) = 0.001, p(Maximal respiration) = 0.0013. *N* = 4. **(G)** NAD^+^ concentration was detected by NAD^+^ production detection kit. *p* = 0.016. *N* = 3. Scale bar, 50 μm. *P ≤ 0.05, **P ≤ 0.01.

To further investigate mitochondrial function, a transmission electron microscope was used, which revealed that the microglial mitochondria were damaged after sevoflurane exposure (Figure 3E). The black arrowhead in Figure 3E indicated accumulated lysosomes in the sevoflurane-treated group, and the mitochondria morphology also appeared to be damaged. However, the quantification of mitochondrial number in Figure 3E showed that there is no significant difference between the control and sevoflurane treatment groups (Supplementary Figure 3). ATP production also decreased after sevoflurane exposure, as tested using an ATP production detection kit (Figure 3F). The staining of JC-1, an indicator of mitochondrial membrane potential, demonstrated that the JC-1 monomers (green staining) were upregulated after sevoflurane treatment, which suggested a decrease in mitochondrial membrane potential (Figures 4A,B). We also performed a seahorse assay to test the mitochondria function and discovered that sevoflurane exposure led to poor mitochondria function (Figure 4C). ATP production declined (Figure 4D) along with a decrease in basal respiration and maximal respiration after sevoflurane exposure (Figures 4E,F). Because NAD^+^ is vital for mitochondrial function, we also analyzed the NAD^+^ content by using an NAD^+^ production detection kit and discovered that NAD^+^ levels decreased after sevoflurane exposure (Figure 4G). This finding indicated that sevoflurane exposure led to mitochondrial dysfunction, poor ATP production, and inadequate NAD^+^ levels, all of which were responsible for the phagocytic dysfunction of microglia.

### NAD supply could alleviate mitochondrial dysfunction and phagocytosis

**FIGURE 5:**
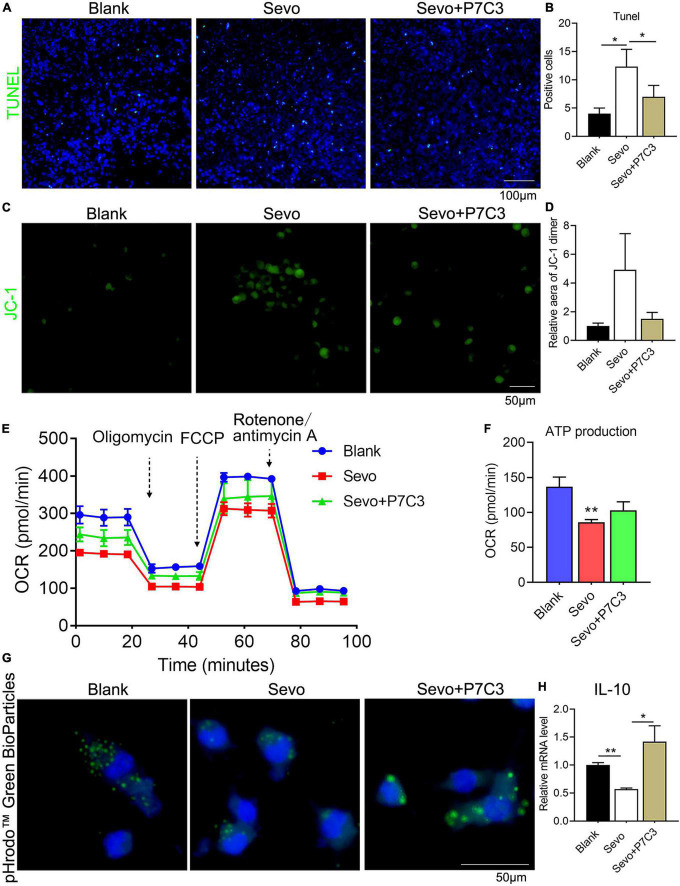
NAD supply ameliorates mitochondrial dysfunction and cell phagocytosis induced by sevoflurane exposure. **(A,B)** Tunel assay of BV2 cell line after P7C3 treatment and the quantification of Tunel-positive cells. p(Blank vs. Sevo) = 0.011, p(Sevo vs. Sevo+P7C3) = 0.027. *N* = 3. **(C,D)** JC-1 staining of BV2 cell line after P7C3 treatment and the quantification of positive-staining area. p(Blank vs. Sevo) = 0.15, p(Sevo vs. Sevo+P7C3) = 0.2. *N* = 3. **(E,F)** The overall mitochondrial metabolism using Seahorse experiment of BV-2 cell line after P7C3 treatment, and ATP production detected by Seahorse experiment. p(Blank vs. Sevo) = 0.0032, p(Sevo vs. Sevo+P7C3) = 0.088. *N* = 3. **(G)** pHrodo™ Green BioParticles staining of BV-2 cell line to determine the phagocytic function after P7C3 treatment. **(H)** qRT-PCR of BV-2 cell line shows the mRNA level of anti-inflammatory factor IL-10. p(Blank vs. Sevo) = 0.005, p(Sevo vs. Sevo+P7C3) = 0.022. *N* = 3. Scale bar, 50 μm. *P ≤ 0.05, **P ≤ 0.01.

To investigate whether NAD^+^ contributed to cell phagocytosis and mitochondrial function, we administered P7C3, an NAD^+^ agonist, to the neonatal mice and the BV2 cell line. The 6 days mice were given 20 mg/kg P7C3 before sevoflurane exposure for 2 h daily for 3 days. A TUNEL assay demonstrated that P7C3 could alleviate cell apoptosis induced by sevoflurane (Figures 5A,B). We also treated the BV2 cell line with 20 μM P7C3 before sevoflurane exposure for 2 h daily for 3 days, and the staining of JC-1 showed that NAD^+^ supply could increase mitochondrial membrane potential (Figures 5C,D). Furthermore, a seahorse assay suggested that P7C3 treatment enhanced mitochondrial function (Figure 5E) and slightly increased ATP production (Figure 5F). P7C3 could also promote cell phagocytosis as demonstrated by increased BioParticles engulfment (Figure 5G). The level of the anti-inflammatory factor IL-10 increased after P7C3 administration (Figure 5H). This finding demonstrated that NAD^+^ supply could decrease the rate of cell apoptosis induced by sevoflurane exposure and promote mitochondrial function and cell phagocytosis, indicating that neurotoxicity caused by microglial phagocytosis dysfunction was moderated by the effects of NAD^+^ on mitochondrial function.

## Discussion

**FIGURE 6:**
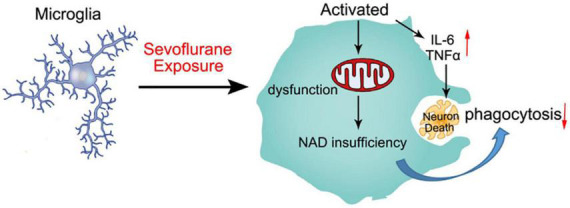
Schematic diagram of sevoflurane-induced neurotoxicity by regulating the mitochondrial function of activated microglia due to NAD insufficiency. Microglia were activated by sevoflurane exposure and secreted proinflammatory factors to impair developing neurons. Mitochondrial function was also damaged after sevoflurane treatment, which could not produce sufficient NAD^+^ to engulf neuron debris. The deficient phagocytosis of microglia resulted in apoptotic neuron accumulation.

Our findings are similar to those clinical studies, that is, developing brains that received sevoflurane repeatedly are more prone to cognitive impairment (Wilder et al., 2009; Flick et al., 2011). According to other reports, neuroinflammation is associated with cognitive dysfunction in humans and animals (Patanella et al., 2010). In our study, we found that anesthesia with 3% sevoflurane for 2 h daily for 3 days in young mice induced microglia activation to secrete proinflammatory factors, including TNFα, IL-6, IL-1α, and IL-1β (Figure 2). Proinflammatory cytokines induce neuron degeneration and neurobehavioral deficits (Sparkman et al., 2005; Barrientos et al., 2006; Schmidt et al., 2006; Liu et al., 2007; Wan et al., 2007). Also, we demonstrated that the mitochondrial function of microglia was obstructed by sevoflurane exposure due to NAD^+^ insufficiency, which led to the poor phagocytosis of activated microglia. The neuron debris could not be engulfed by the activated microglia, which resulted in apoptotic neuron accumulation (Figure 6).

Anesthesia with sevoflurane could activate microglia and disrupt brain homeostasis by influencing the expression of proinflammatory factors. Activated microglia are characterized by enhancing the release of proinflammatory cytokines, a process that has been demonstrated by a number of proofs. Moreover, we found that sevoflurane exposure blocked the anti-inflammatory state of microglia, which was detrimental to the phagocytic function of microglia. Phagocytic dysfunction resulted in the inability of activated microglia to engulf degenerated neurons and debris, which in turn aggravated neurodegeneration. It is important to clear neuronal debris to maintain CNS function, because neuronal debris triggers neuroinflammation that leads to mitochondrial dysfunction of microglia. Studies have demonstrated that the damaged and fragmented microglial mitochondria are released into the neuronal milieu and impair neuronal function (Joshi et al., 2019). However, the mechanism through which mitochondria release debris to the extracellular space is still unknown. Some reports have argued that the mitochondrial permeability transition may depend on the release of extracellular vesicles (van Niel et al., 2018), which may originate from astrocytes and microglia (Hooper et al., 2012; Glebov et al., 2015). However, another study provides evidence for the opinion that direct exocytosis may contribute to this debris release of mitochondria (Hessvik and Llorente, 2018). Inadequate energy in turn promotes the proinflammatory state of microglia and other macrophages (Minhas et al., 2021).

The microglial mitochondria are critical for brain homeostasis, because they generate energy to maintain neuronal development. Studies have demonstrated that functional mitochondria could transfer from microglia and astrocytes to neurons to protect neurons from injury (Hayakawa et al., 2016; Joshi et al., 2019). The phagocytic function of microglia to remove dysfunctional or unnecessary structures, such as degenerated neurons, is deemed neuroprotective (Lemke, 2019). Mitochondria play a major role not only in regulating metabolism but also in mediating macrophage function. The dynamics of mitochondrial morphology are mediated by mitofusin-1(Mfn1), Mfn2, and optic atrophy type 1. Macrophages with Mfn2 deletion exhibit fragmented mitochondria and poor phagocytic function (Tur et al., 2020), which suggests that mitochondria are indispensable for the phagocytic function of macrophages.

NAD^+^ deficiency suppresses mitochondrial respiration and impairs phagocytosis and the resolution of inflammation. Accelerated NAD^+^ generation in macrophage restored oxidative phosphorylation (Minhas et al., 2019). Recent studies have clarified that cellular metabolism can control macrophage polarization states, which regulate proinflammatory responses (Jha et al., 2015), and that the inhibition of *de novo* NAD^+^ synthesis leads to the accumulation of proinflammatory factors and suppression of anti-inflammatory factors (Minhas et al., 2019). Our results also demonstrate that NAD^+^ synthesis is critical for mitochondrial metabolism and also affects microglia polarization. NAD^+^ restoration could not only mitigate the mitochondrial dysfunction and phagocytic malfunction of microglia but also alleviate cell apoptosis induced by sevoflurane exposure. We should further investigate the underlying mechanism of how the mitochondrial function mediated by NAD^+^ affects the phagocytic function of microglia.

Calmodulin-dependent protein kinase II (CaMKII) is associated with neuronal damage that can affect learning and memory (Silva et al., 1992), and sevoflurane exposure can increase CaMKII expression, which affects mitochondrial potential and results in neuronal damage (Han et al., 2020). The study has demonstrated that CaMKII is a critical mediator of Ca^2+^/CaM signaling (Griffith, 2004). The CaMKII inhibitor KN93 can ameliorate cell apoptosis induced by inadequate NAD^+^ concentration (Takeuchi and Yamamoto, 2015). *In vitro* study has implied that NAD could be phosphorylated to yield NADP using NAD kinase (NADK) enzymes (Labesse et al., 2002). The addition of Ca^2+^ and CaM enhances NADK activity and results in NADP production (Love et al., 2015), which suggests a decrease in substrate NAD^+^ production. In the future, we should study whether CaMKII is involved in NAD^+^ affection of regulating microglia function, and how NAD^+^ mediates the mitochondrial function of microglia after sevoflurane exposure.

## Data availability statement

The datasets presented in this study can be found in online repositories. The names of the repository/repositories and accession number(s) can be found in the article/Supplementary material.

## Ethics statement

The animal study was reviewed and approved by the Welfare and Ethics Committee of Experimental Animal Center of Zhengzhou University.

## Author contributions

RZ and JZ supported the research conception and design. RZ wrote the draft of the manuscript. SZ, NL, NF, MM, YY, and MS performed the material preparation, data collection, and analysis. All authors read and approved the final manuscript and commented on previous versions of the manuscript.

## Supplementary material

Supplementary Figure 1 Sevoflurane exposure declined the neuron numbers. **(A,B)** Immunofluorescence staining and quantification of neuron marker NeuN. *p* = 0.039. *N* = 3. Scale bar, 100μm.

Supplementary Figure 2 TNFα ELISA experiment showed that TNFα concentration increased after sevoflurane exposure. *p* = 0.001. *N* = 6.

Supplementary Figure 3 The mitochondrial quantification of the EM image showed that there’s no significant difference in mitochondrial number between the control and sevoflurane groups. *p* = 0.62. *N* = 5.
