# Supplementary figures and images for: Sevoflurane exposure induces neurotoxicity by regulating mitochondrial function of microglia due to NAD insufficiency
Source: Front Cell Neurosci. 2022 Sep 21;16:914957. doi: 10.3389/fncel.2022.914957 (PMC9532507; doi:10.3389/fncel.2022.914957)

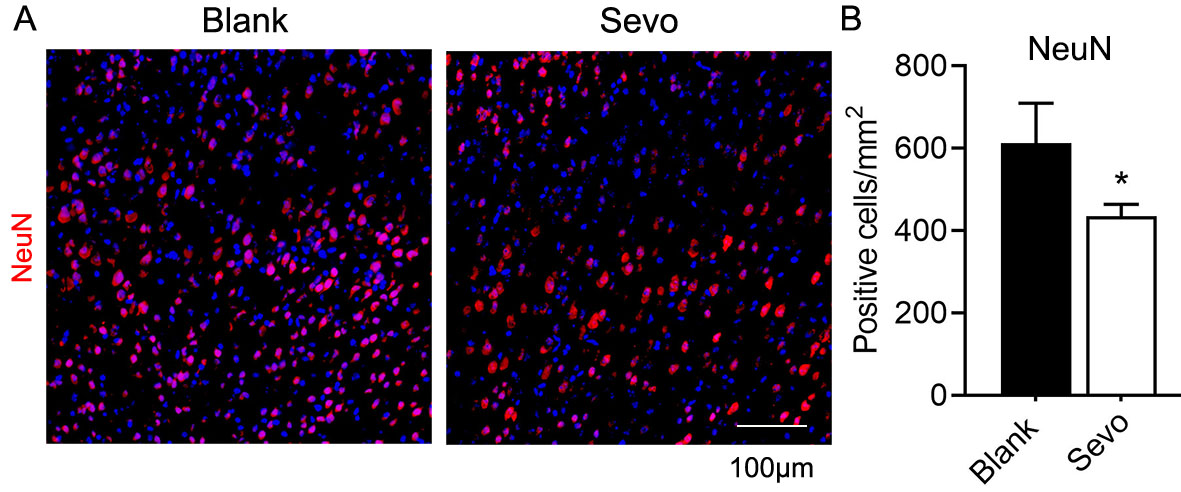

Supplement: Supplementary Figure 1 — Sevoflurane exposure declined the neuron numbers. (A,B) Immunofluorescence staining and quantification of neuron marker NeuN. p = 0.039. N = 3. Scale bar, 100μm. [file Image_1.JPEG]

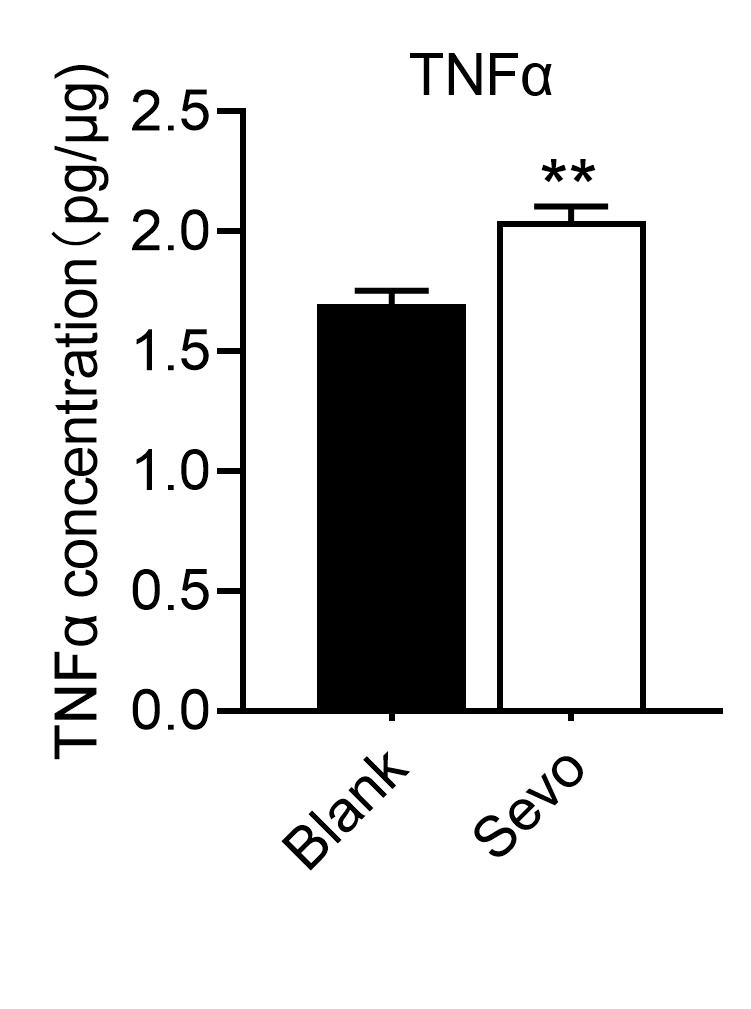

Supplement: Supplementary Figure 2 — TNFα ELISA experiment showed that TNFα concentration increased after sevoflurane exposure. p = 0.001. N = 6. [file Image_2.JPEG]

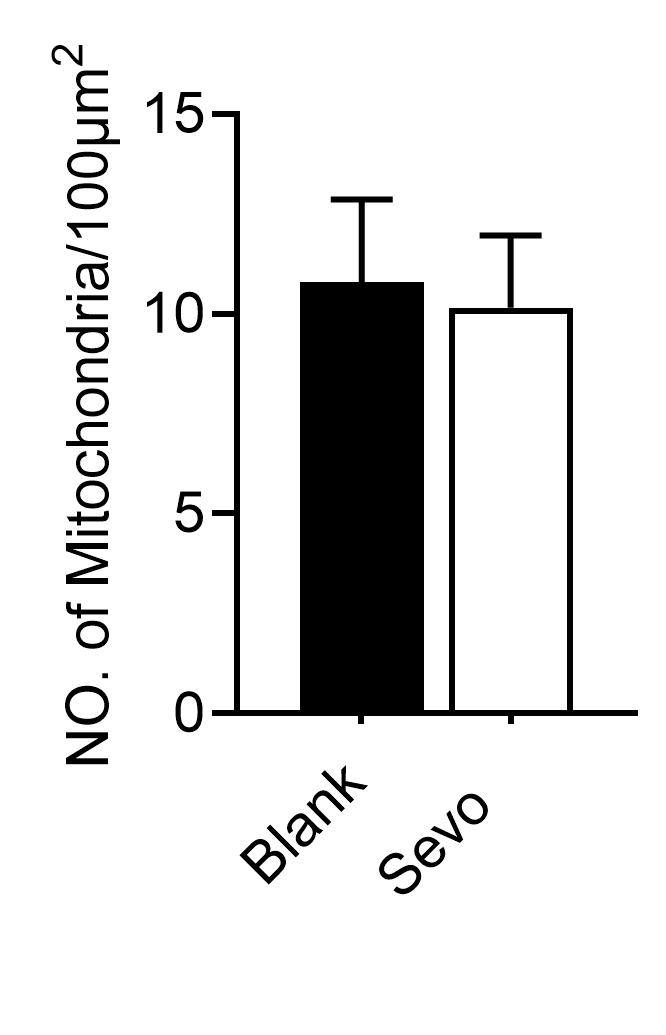

Supplement: Supplementary Figure 3 — The mitochondrial quantification of the EM image showed that there’s no significant difference in mitochondrial number between the control and sevoflurane groups. p = 0.62. N = 5. [file Image_3.JPEG]
